# SB203580 increases G-CSF production via a stem-loop destabilizing element in the 3’ untranslated region in macrophages independently of its effect on p38 MAPK activity

**DOI:** 10.1186/s12929-016-0221-z

**Published:** 2016-01-16

**Authors:** Shwu-Fen Chang, Huai-Ci Li, Yu-Pei Huang, Wen-Ju Tasi, Yuan-Yi Chou, Shao-Chun Lu

**Affiliations:** Graduate Institute of Medical Sciences, College of Medicine, Taipei Medical University, Taipei, Taiwan; Department of Biochemistry and Molecular Biology, College of Medicine, National Taiwan University, Room 810, No.1, Jen Ai Road Section 1, Taipei, Taiwan

**Keywords:** G-CSF, mRNA, p38 MAPK, Pyridinyl imidazole compounds, SB203580, 3’ untranslated region

## Abstract

**Background:**

Granulocyte-colony stimulating factor (G-CSF) is a major regulator of the production and survival of neutrophils. Regulation of G-CSF expression is complex and occurs at both transcription and post-transcription levels. Two distinct types of cis-acting elements in the 3’ untranslated region (3’UTR) of G-CSF mRNA have been identified as destabilizing elements; these consist of adenylate uridylate-rich elements (AUREs) and a stem–loop destabilizing element (SLDE). Regulation of the stability of mRNA by p38 mitogen-activated protein kinase (MAPK) has been indicated to be linked to AUREs in the 3’UTR. However, whether p38 MAPK is involved in the regulation of the stability of G-CSF mRNA has not been elucidated. This study investigated the effect of SB203580, an inhibitor of p38 MAPK, on the lipopolysaccharide-induced G-CSF expression in macrophages at the post-transcription level.

**Results:**

Our study showed surprising results that SB203580 augmented the lipopolysaccharide-induced increase in the G-CSF mRNA levels in RAW264.7 mouse macrophages, mouse bone marrow-derived macrophages and in THP-1 human macrophages. This effect was also seen in p38α MAPK knockdown RAW264.7 cells, showing that it was not due to inhibition of p38 MAPK activity. In the presence of actinomycin D, the decay of G-CSF mRNA was slower in SB203580-treated cells than in control cells, showing that SB203580 increased the stability of G-CSF mRNA. Reporter genes containing luciferase with or without the 3’UTR of G-CSF were constructed and transfected into RAW264.7 cells and the results showed that the presence of the 3’UTR reduced the luciferase mRNA levels and luciferase activity. Furthermore, SB203580 increased the luciferase mRNA levels and activity in RAW264.7 cells transfected with the luciferase reporter containing the 3’UTR, but not in cells transfected with the luciferase reporter without the 3’UTR. Mutations of the highly conserved SLDE in the 3’UTR abolished these effects, showing that the SLDE was essential for the SB203580-induced increase in the stability of mRNA.

**Conclusions:**

SB203580 increases G-CSF expression in macrophages by increasing the stability of G-CSF mRNA via its 3’UTR, and the effect was not due to its inhibition of p38 MAPK activity. The results of this study also highlight a potential target for boosting endogenous production of G-CSF during neutropenia.

## Background

Granulocyte colony-stimulating factor (G-CSF), a glycoprotein, stimulates the proliferation of neutropenic progenitor cells and their differentiation to granulocytes and also activates mature neutrophils [1]. Production of neutrophils is critical for innate host defense against microbial pathogens and is tightly controlled. G-CSF is primarily secreted by monocytes and macrophages, but is also produced by endothelial cells, fibroblasts, and bone marrow stromal cells [2]. Transcriptional control of G-CSF expression has been intensively studied. Binding elements for nuclear factor- kappaB (NF-κB), nuclear factor for IL-6 (NF-IL6), and octamer transcription factors in the G-CSF promoter are highly conserved and are required for endotoxin-mediated induction of G-CSF transcription in macrophages [3]. However, post-transcriptional regulation of the stability of G-CSF mRNA has been less well studied. Two distinct types of cis-acting elements in the 3’ untranslated region (3’UTR) of G-CSF mRNA have been identified as destabilizing elements; these consist of adenylate uridylate-rich elements (AUREs), with the core sequence AUUUA, and a stem–loop destabilizing element (SLDE) [4]. AUREs are the best characterized mRNA destabilizing elements, and have been identified in the 3’UTR of many transiently expressed cytokine mRNAs, including those for tumor necrosis factor-alpha (TNF-α), interleukin-1 beta (IL-1β), cyclooxygenase-2 (COX-2), and interferon-γ; however, they are also known to increase the stability of mRNA in macrophages treated with lipopolysaccharide (LPS) [5]. In NIH 3 T3 cells, the SLDE in the 3’UTR of G-CSF mRNA operates by a mechanism different from that used by AUREs, as mRNA degradation directed by AUREs is inhibited by calcium flux generated by a calcium ionophore, whereas that directed by SLDE is not [4, 6].

SB203580 is originally identified as an inflammatory cytokine synthesis inhibitor and subsequently found to be a selective inhibitor of p38α and p38β mitogen-activated protein kinases (MAPKs) [7]. SB203580 inhibits the catalytic activity of p38 MAPK by competitively binding to the ATP pocket and is widely used to elucidate the roles of p38 MAPK in a wide range of biological systems. Genetic deletion of one form, p38β MAPK, in mice does not affect LPS-induced production of cytokine [8], suggesting that p38β MAPK is not involved in the immune and inflammatory response, while the other form, p38α MAPK, is known to regulate synthesis of cytokines by increasing mRNA half-life [7]. Inhibition of p38 MAPK activity by SB203580 in LPS-treated THP-1 macrophages leads to rapid degradation of several AURE containing mRNAs such as COX-2, IL-1β, IL-6, and MAP2K6 [9]. However, in contrast to this decrease in these mRNA levels in response to SB203580 treatment, in the present study, we observed that SB203580 increased G-CSF mRNA and protein levels in LPS-treated RAW264.7 and THP-1 macrophages. Since AUREs increase the stability of COX-2, IL-1β, IL-6, and MAP2K6 mRNAs through a p38 MAPK-dependent pathway [9], it is possible that the SB203580-induced increase in the G-CSF mRNA levels is mediated by AUREs and might be mediated by the other destabilizing element, SLDE. Here, we show that the SB203580-induced increase in the G-CSF mRNA levels does not involve p38α MAPK and that the SLDE in the 3’UTR is essential for SB203580-induced G-CSF mRNA stabilization.

## Methods

### Materials

Dulbecco’s modified Eagle’s medium (DMEM), RPMI 1640 medium, and fetal bovine serum (FBS) were obtained from Hyclone Laboratories (Logan, UT, USA). TRIzol reagent and penicillin/streptomycin were obtained from GibcoBrl/LifeTechnologies (Rockville, MD, USA). LPS from *Escherichia coli* (serotype 0111:B4) and actinomycin D, a transcription inhibitor, were purchased from Sigma-Aldrich (St. Louis, MO, USA). Mouse macrophage colony-stimulating factor (M-CSF) was obtained from PeproTech (Rocky Hill, NJ, USA.). Mouse monoclonal anti-β-actin antibody was purchased from Chemicon (Temecula, CA, USA), p38α MAPK mouse mAb and rabbit polyclonal anti-mouse phospho-p38 antibodies were from Cell Signaling Technology (Danvers, MA, USA), and peroxidase-conjugated anti-rabbit or anti-mouse IgG antibodies were from Amersham-Pharmacia Biotech (Piscataway, NJ, USA). The pGL3-Basic and phRLTK reporter plasmids, Dual-Luciferase® Reporter Assay System, and MMLV reverse transcriptase were from Promega (Madison, WI, USA). The SuperFect Transfection reagent was purchased from Qiagen (Hilden, Germany). The mouse G-CSF Quantikine ELISA kit was from R&D Systems (Minneapolis, MN, USA). The pharmacological p38 MAPK inhibitors SB203580, SB202190, SB239063, PD169316, and SKF86002 and the inactive SB203580 analog SB202474 were purchased from Calbiochem (San Diego, CA, USA) and dissolved in dimethyl sulfoxide (DMSO).

### Cell culture

RAW264.7, a murine macrophage cell line was cultured in DMEM supplemented with 10 % FBS, 4 mM glutamine, 100 U/ml of penicillin, and 100 μg/ml of streptomycin at 37 °C in 5 % CO_2_ as described previously [10]. THP-1, a human acute monocytic leukemia cell line was cultured in RPMI 1640 medium containing 10 % FBS, 100 U/ml of penicillin, and 100 μg/ml of streptomycin and induced to differentiate to macrophages using 160 nM phorbol 12-myristate-13-acetate as described previously [10]. Mouse bone marrow-derived macrophages (BMDMs) were differentiated from bone marrow cells as described previously [11]. The procedures for the use of mouse bone marrow cells were reviewed and approved by the National Taiwan University Institutional Animal Care and Use Committee. Briefly, mouse bone marrow cells were aspirated from the tibias and femurs of 16- to 20 week-old C57BL/6 J mice and cultured in a humidified incubator in an atmosphere of 5 % CO_2_ at 37 °C in complete culture medium containing 25 % L-929 cell conditioned medium on bacteriologic Petri dishes for 7 days. After trypsinization, the cells were counted and seeded at 1×10^6^ cells/ml in complete culture medium containing 10 ng/ml of M-CSF overnight before further experiments. L-929 cells conditioned medium was prepared by incubating 5×10^5^ L-929 cells in a T-150 flask (Corning, NY, USA) for 1 week in complete culture medium (DMEM supplemented with 10 % FBS, 2 mM L-glutamine, 1 mM sodium pyruvate, 100 U/ml of penicillin, and 100 μg/ml of streptomycin).

### Effect of p38 MAPK inhibitors on G-CSF mRNA and protein levels

G-CSF mRNA and protein levels were compared in cells treated with 0.1 % DMSO (vehicle) and cells treated with p38 MAPK inhibitors (10 μM unless otherwise specified) for the indicated time or in cells pretreated for 30 min with DMSO or p38 MAPK inhibitor, followed by addition of 100 ng/ml of LPS and further incubation for the indicated time.

### Quantification of G-CSF protein in culture medium

The concentration of G-CSF in the culture medium from RAW264.7 cells was measured using a mouse G-CSF Quantikine ELISA kit (R & D Systems, Minneapolis, MN, USA) according to the manufacturer’s instructions.

### RNA isolation and mRNA analyses

Total cellular RNA was isolated from cells using TRIzol® reagent (Invitrogen, Carlsbad, CA, USA), and RNA concentrations determined from the absorbance at 260 nm. First-strand cDNA was synthesized from total RNA using MMLV reverse transcriptase (RT) with oligo-dT as primer. Levels of G-CSF and GAPDH mRNA were determined either by semi-quantitative PCR (RT-PCR) or by real-time quantitative PCR (RT-qPCR) on an iCycler instrument (BioRad, Hercules, CA, USA) as indicated. The specific primer sets were designed using Beacon designer 5.0 (Premier Biosoft International, Palo Alto, CA, USA) and are listed in Table 1. The amplified DNA fragments were confirmed by sequencing. The fold induction of mRNA expression was quantified by densitometry (RT-PCR) or by the ΔΔCt-method (RT-qPCR) as described previously [12].

**Table 1:** Equences of the primers used in this study

Primers for RT-PCR	
Primers	Sequence
mouse G-CSF	Forward 5’-CTCAACTTTCTGCCCAGAGG-3’
	Reverse 5’-CTGGAAGGCAGAAGTGAAGG-3’
human G-CSF	Forward 5’-CACTCTGGACAGTGCAGGAAG-3’
	Reverse 5’-CGACACCTCCAGGAAGCTCTG-3’
mouse IL-1β	Forward 5’-GACCTTCCAGGATGAGGACA-3’
	Reverse 5’-AGGCCACAGGTATTTTGTCG-3’
human & mouse GAPDH	Forward 5’-AAAGGATCCACTGGCGTCTTCACCACC-3’
	Reverse 5’-GAATTCGTCATGGATGACCTTGGCCAG-3’
Primers for RT-qPCR	
Primers	Sequence
mouse G-CSF	Forward 5’-TTGGCAACATCCAGCTGAAG-3’
	Reverse 5’-GCAGGCTCTATCGGGTATTTCC-3’
human G-CSF	Forward 5’-TCCCCATCCCATGTATTTATCT-3’
	Reverse 5’-AACTCAGAAATGCAGGGAAGGA-3’
human & mouse GAPDH	Forward 5’-GGCATTGTGGAAGGGCTCAT-3’
	Reverse 5’-GACACATTGGGGGTAGGAACAC-3’
*Photinus* luciferase	Forward 5’-GCCTGAAGTCTCTGATTAAGT-3’
	Reverse 5’-ACACCTGCGTCGAAGA-3’
*Renilla* luciferase	Forward 5’-GCAAGGGTTGGTCGTGAGG-3’
	Reverse 5’-TCATCCGTTTCCGTTCTG-3’
Primers for plasmid construction	
Primers	Sequence
mouse GAPDH promoter	Forward 5’-TTACGCGTGATGATGGAGGACGTGATG-3’
	Reverse 5’-ATCAGATCTGCAGGAGAAGAAAATGAG-3’
mouse G-CSF 3’UTR	Forward 5’-AGTCTAGACCTGAGCAGAAAGCCCTTTCC-3’
	Reverse 5’-AGTCTAGAGGAACACCACACTTTATTATCCGCA-3’

The underlined sequences are restriction enzyme sites created to facilitate cloning

### Plasmid construction

A 107 bp (-81 to +26) fragment of the GAPDH promoter was PCR-amplified from mouse genomic DNA using the primers listed in Table 1 and cloned into the *Mlu*I/*Bgl*II sites at the 5’ end of the luciferase gene in the pGL3-basic reporter plasmid to generate the plasmid Gp-LUC. The mouse G-CSF mRNA 3’UTR fragment from nucleotide 691 to 1363 was PCR-amplified from cDNA generated from RAW264.7 cell total RNA using the primers listed in Table 1 and was cloned into *Xba*I site of the Gp-LUC reporter plasmid to obtain the Gp-LUC-3’UTR reporter plasmid. Site-directed mutagenesis to generate three Gp-LUC-3’UTR SLDE mutants, SLDE M1, M2, and M3, was performed by PCR using mutant oligonucleotides. All constructs were verified by sequencing.

### Reporter gene activity assay

To evaluate the effects of the G-CSF 3’UTR on luciferase activity, 0.75 μg of Gp-LUC, Gp-LUC-3’UTR, or an SLDE mutant reporter plasmid was mixed with 0.75 μg of phRLTK plasmid, then the mixture was transiently transfected into RAW264.7 cells. Briefly, RAW264.7 cells were plated in 24-well plates and cultured overnight before transfection with Superfect reagent as described previously [10]. Four hours after transfection, in most cases, the cells were either left untreated or were pretreated with DMSO or SB03580 for 30 min before and during incubation with 100 ng/ml of LPS for 20 h, while, in some cases, the transfected cells were incubated with DMSO or SB03580 for 20 h without subsequent addition of LPS. At 24 h after transfection, *Photinus* and *Renilla* luciferase activities in the cell lysates were assayed using the Dual-Luciferase Reporter Assay System (Promega, Madison, WI, USA), and the light intensity produced by *Photinus* luciferase (test plasmid) was normalized to that produced by *Renilla* luciferase (control plasmid). In some experiments, *Photinus* luciferase and *Renilla* luciferase mRNA levels were determined by RT-qPCR using the specific primers listed in Table 1.

### Knockdown of p38 by RNA interference

The pLKO.1-short hairpin RNA (shRNA) plasmids encoding an shRNA targeting firefly luciferase (5’–CAAATCACAGAATCGTCGTAT–3’) or one targeting mouse p38α (5’–CCTCTTGTTGAAAGATTCCTT–3’) were obtained from the National RNAi Core Facility at the Academia Sinica, Taiwan, and were transfected, together with pMD.G and pCMV delta8.91, into a HEK293T packaging cell line (National RNAi Core Facility, Taiwan) using the calcium phosphate method, and culture supernatants containing lentivirus were collected from the medium 60 h later. For knockdown experiments, RAW264.7 cells were transduced for 24 h with the collected virus supernatants in the presence of polybrene (8 μg/ml), then infected cells were selected with puromycin (10 μg/ml) for 10 days.

### Western blot analysis

Cells were washed with ice-cold phosphate-buffered saline and lysed with RIPA buffer (20 mM Tris-HCl, pH 7.5, 150 mM NaCl, 5 mM EDTA, 0.5 % NP-40, 0.5 % Triton X-100, 0.1 % SDS, 1 mM NaF, 1 mM PMSF, 1 mM Na_3_VO_4_, and 1 μg/ml of leupeptin), then the lysates were centrifuged at 500 × g for 10 min at 4 °C and samples of the supernatants (20 μg of protein/lane) were separated by SDS-PAGE on a 10 % gel and transferred to a PVDF membrane, which was then blocked overnight at 4 °C with blocking buffer (10 mM Tris-HCl, pH 8.0, 0.15 M NaCl, 0.1 % Tween 20, and 5 % fat-free milk). The blots were then incubated for 1 h at room temperature with 0.5 μg/ml of mouse monoclonal anti-p38α antibody, rabbit polyclonal anti-phospho-p38 antibodies, or mouse monoclonal anti-β-actin antibody and for 40 min at room temperature with peroxidase-conjugated anti-rabbit or anti-mouse IgG antibodies, then bound antibody was detected using an improved chemiluminescence detection system (NEN, Boston, MA, USA). Protein concentrations were determined by the Bradford method (DC Protein Assay, Bio-Rad, Hercules, CA, USA).

### Statistical analysis

Results are shown as the mean ± SD. Differences between means were evaluated using Student’s *t* test and were considered significant at *p* <0.05.

## Results

### SB203580 increases G-CSF protein and mRNA levels in LPS-stimulated RAW264.7 cells, BMDMs and THP-1 cells

**Fig. 1:**
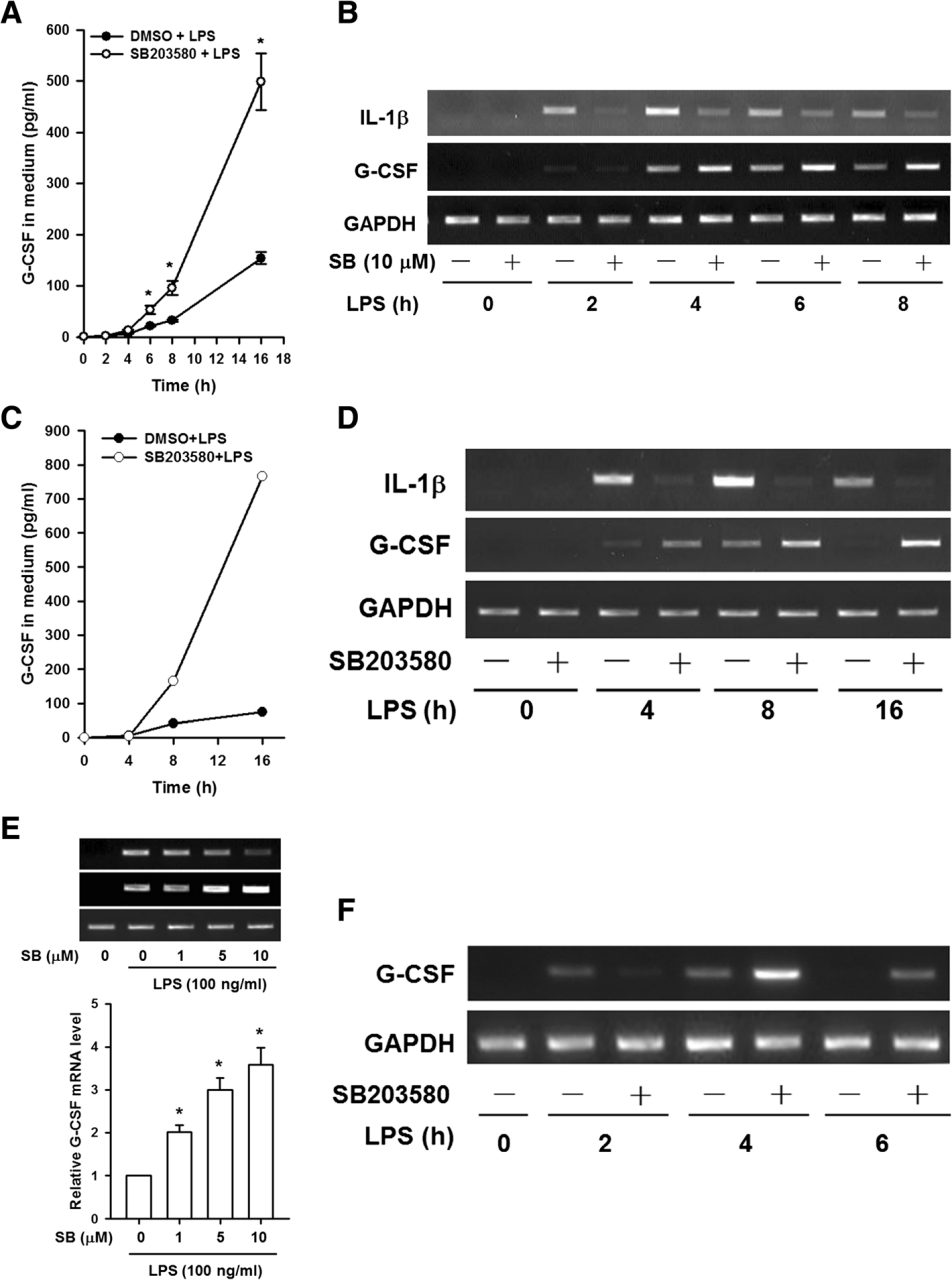
SB203580 enhances G-CSF expression in LPS-treated macrophages. RAW264.7 cells (**a** and **b**) and BMDMs (**c** and **d**) were incubated with DMSO (vehicle) or 10 μM SB203580 for 30 min, then 100 ng/ml of LPS was added and incubation continued for 0 to 16 h, then G-CSF protein levels in the medium were measured by ELISA (**a** and **c**) and G-CSF and IL-1β mRNA levels were determined by RT-PCR and analyzed by gel electrophoresis (**b** and **d**). (**e**) RAW264.7 cells were incubated with DMSO or 1, 5, or 10 μM SB203580 for 30 min, then LPS (100 ng/ml) was added and incubation continued for 6 h, then G-CSF and IL-1β mRNA levels were determined by RT-PCR (*upper panel*). Levels of G-CSF mRNA were determined by RT-qPCR (*lower panel*), normalized to GAPDH mRNA levels, and expressed relative to the value in the cells treated only with LPS, set as 1. (**f**) THP-1 macrophages were pretreated with DMSO or 10 μM SB203580 for 30 min, then LPS or PBS was added and incubation continued for 0 to 6 h. Total RNA was then isolated and levels of G-CSF and GAPDH (internal control) mRNA determined by RT-PCR and analyzed by gel electrophoresis. The results in (**a**) and (**e**) are the mean ± SD for three independent experiments. *p < 0.05, compared to the group treated only with LPS. (**d**) The data shown in (**b**) and (**f**) are typical of the results obtained in three independent experiments. The data shown in (**c**) and (**d**) are typical of the results obtained in two independent experiments

The effect of SB203580, a p38 MAPK inhibitor, on G-CSF protein and mRNA levels in LPS-stimulated macrophages was examined by incubating RAW264.7 cells with SB203580 or DMSO for 30 min before and during treatment with LPS for various times, then G-CSF protein and mRNA levels were measured by ELISA or RT-PCR, respectively. The results showed that the G-CSF protein levels in the medium (Fig. 1a) and the G-CSF mRNA levels (Fig. 1b) were low in untreated RAW264.7 cells, but increased considerably after, respectively, 6 h or 4 h of LPS treatment and that SB203580 pretreatment resulted in a further significant increase in both G-CSF protein and mRNA levels. Whereas IL-1β mRNA levels were increased after 2 h and reached its peak at 4 h of LPS treatment and that SB203580 pretreatment resulted in a significant decrease in IL-1β mRNA levels (Fig. 1b). The effect of SB203580 on LPS-induced G-CSF expression was also evaluated in mouse BMDMs. As in RAW264.7 cells, G-CSF protein (Fig. 1c) and mRNA (Fig. 1d) were undetectable in untreated BMDMs, but levels of both were increased after, respectively, 8–16 h or 4–8 h of LPS treatment, and SB203580 pretreatment resulted in a further significant increase in the presence of LPS that lasted for at least 16 h. Whereas IL-1β mRNA were upregulatd by LPS and reached its peak at 8 h of LPS treatment and that SB203580 pretreatment resulted in a significant decrease in IL-1β mRNA levels (Fig. 1d). These results were unexpected. To confirm the effect of SB203580 on the G-CSF mRNA levels, RAW264.7 cells were incubated with 0, 1, 5, or 10 μM SB203580 for 30 min before and during treatment with 100 ng/ml of LPS for 6 h, then the G-CSF mRNA levels were measured by RT-PCR and RT-qPCR and the results showed that the G-CSF mRNA levels increased in a dose-dependent manner in response to SB203580 treatment (Fig. 1e). In contrast, IL-1β mRNA levels decreased in a dose-dependent manner in response to SB203580 treatment (Fig. 1e, upper panel).

The effects of SB203580 and LPS on the G-CSF mRNA levels were also evaluated in THP-1 human macrophages, and as with mouse macrophages, SB203580 enhanced the LPS-induced increase in the G-CSF mRNA levels, the effect being maximal after 4 h of LPS treatment (Fig. 1f). Taken together, Fig. 1 shows that SB203580 enhances the LPS-induced increase in G-CSF protein and/or mRNA levels in both mouse and human macrophages.

### SB203580 enhancement of the LPS-induced increase in the G-CSF mRNA levels is not due to p38 MAPK inhibition

**Fig. 2:**
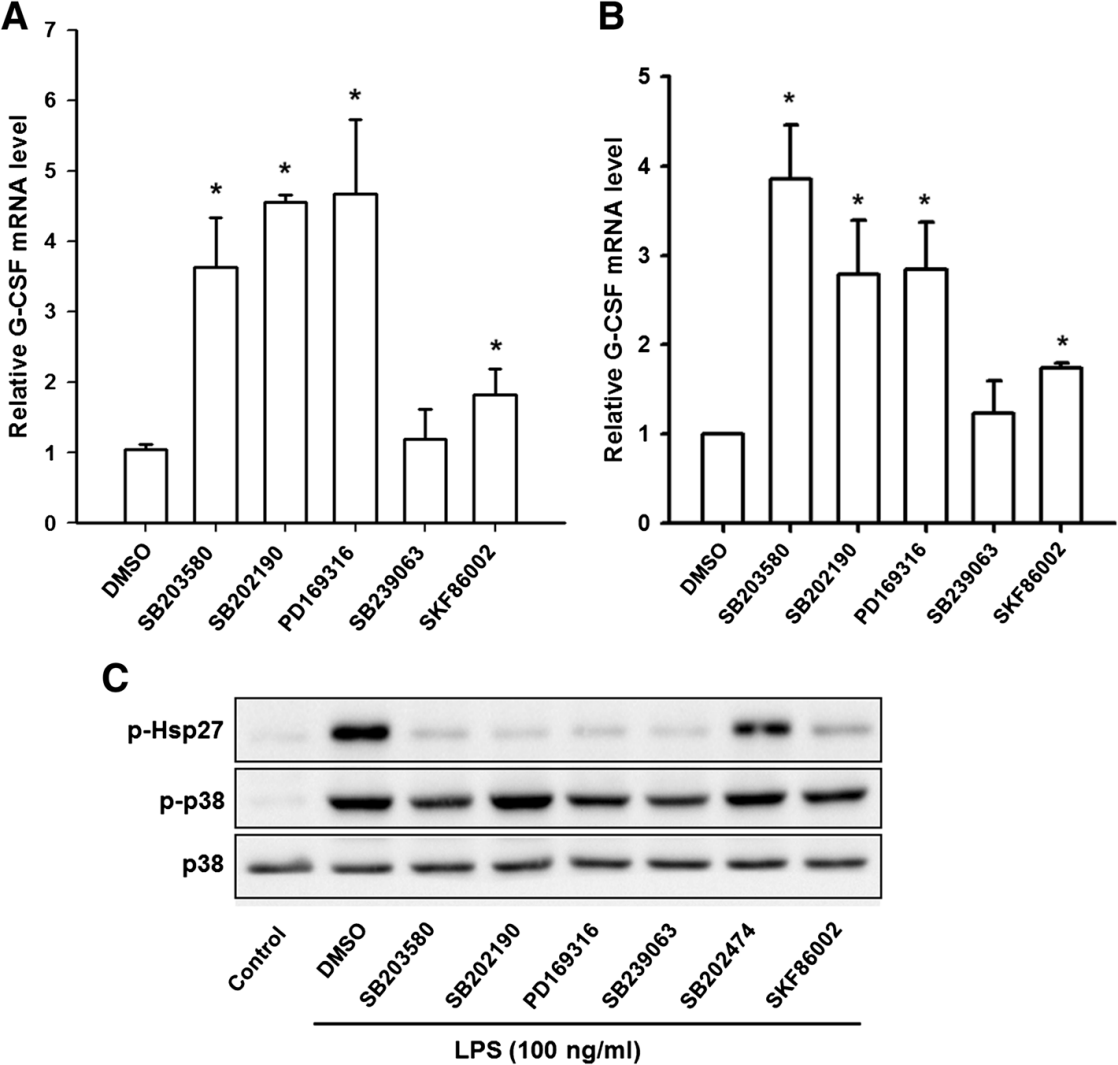
Effects of various p38 MAPK inhibitors on LPS-induced G-CSF mRNA in RAW264.7 and THP-1 macrophages. RAW264.7 cells (**a**) and THP-1 (**b**) were pretreated for 30 min with DMSO or 10 μM SB203580, SB202190, PD169316, SB239063, or SKF86002, then 100 ng/ml of LPS was added and incubation continued for 6 h, then the G-CSF mRNA levels were determined by RT-qPCR, normalized to GAPDH mRNA levels, and expressed relative to the value in the DMSO control, set as 1. The values are the mean ± SD for three separate experiments. *p < 0.05, compared to the DMSO-treated cells. (**c**) THP-1 macrophages were left untreated (*left lane*) or were pretreated for 30 min with DMSO or 10 μM SB203580, SB202190, PD169316, SB239063, or SKF86002, then 100 ng/ml of LPS was added and incubation continued for 30 min, then levels of phosphorylated HSP27, phosphorylated p38 MAPK, and total p38 MAPK were determined by Western blotting

**Fig. 3:**
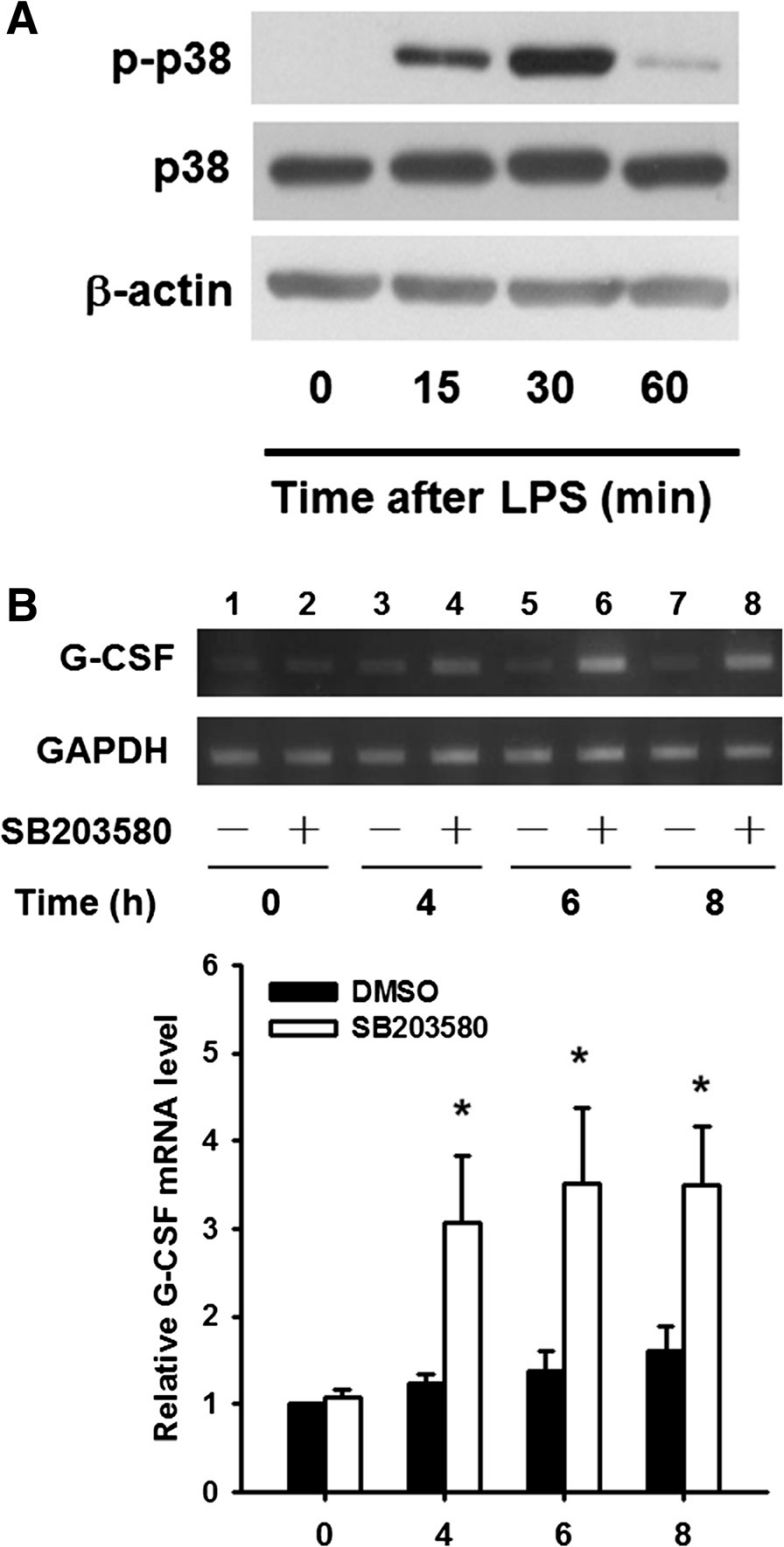
SB203580 induces G-CSF expression in RAW264.7 cells in the absence of LPS stimulation. **a** RAW264.7 cells were left untreated (lane 1) or were incubated with LPS for 15 to 60 min (lanes 2–4), then phosphorylated p38, total p38, and β-actin levels were analyzed by Western blotting. **b** RAW264.7 cells were incubated with DMSO or 10 μM SB203580 for 0 to 8 h, then levels of G-CSF and GAPDH (internal control) mRNA were determined by RT-PCR and analyzed by gel electrophoresis (*upper panels*). The G-CSF mRNA levels were normalized to those for GAPDH mRNA and expressed relative to the value in the DMSO-treated cells at 0 h (relative value = 1) (*lower panels*). The results are the mean ± SD for three independent experiments. *p < 0.05, compared to the DMSO-treated cells

**Fig. 4:**
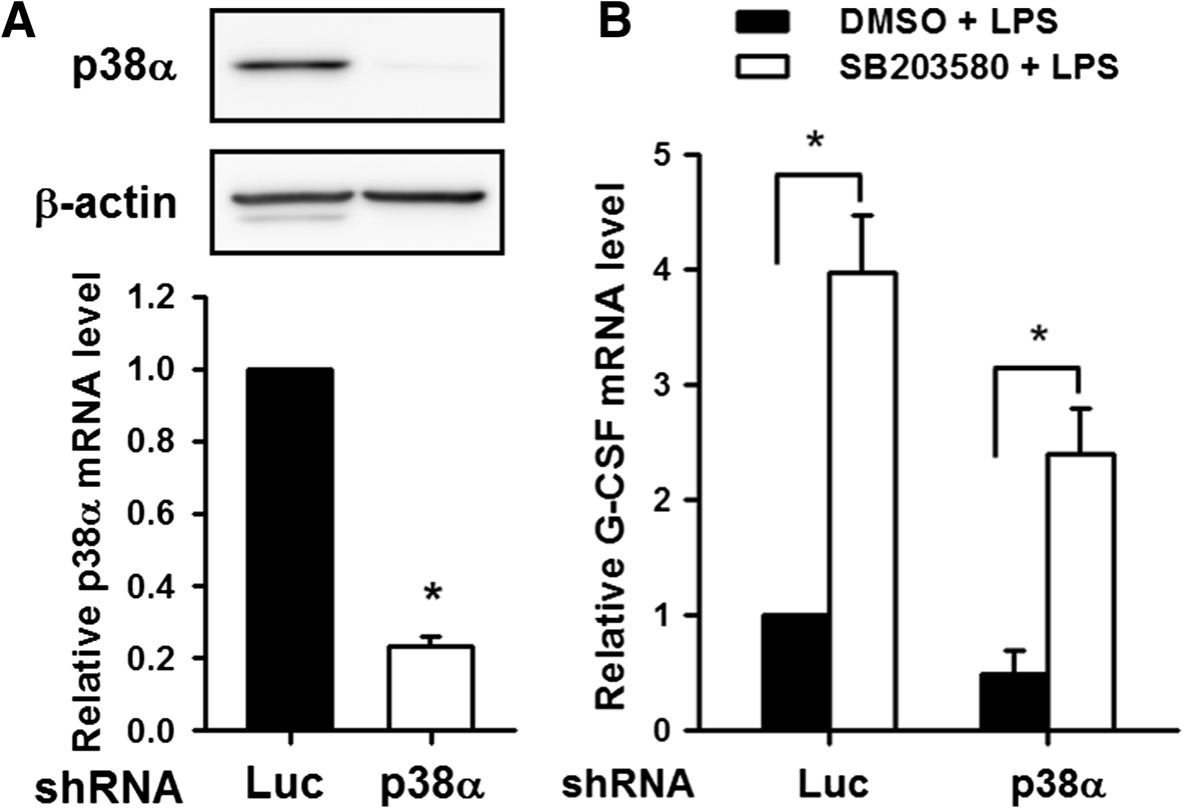
SB203580 enhances the LPS-induced increase in G-CSF expression in p38α MAPK knockdown RAW264.7 macrophages. **a** RAW264.7 cells were infected with either lentivirus carrying specific shRNA for p38α MAPK or lentivirus carrying luciferase shRNA (Luc) and selected with puromycin (5 μg/ml) for 3 days, then p38α MAPK and β-actin protein levels in the cells were determined by Western blotting (upper panel), and mRNA levels were determined by RT-qPCR (lower panel). **b** Control knockdown (Luc) and p38α MAPK knockdown cells were pretreated for 30 min with DMSO or 10 μM SB203580, then 100 ng/ml of LPS was added and incubation continued for 6 h, then the G-CSF mRNA levels were determined by RT-qPCR, normalized to GAPDH mRNA levels, and expressed relative to the value for the appropriate DMSO control, set as 1. The results are the mean ± SD for three independent experiments. *p < 0.05, compared to the corresponding cells not treated with SB203580

To test whether other p38 MAPK inhibitors also enhanced the LPS-induced increase in the G-CSF mRNA levels, RAW264.7 and THP-1 macrophages were pretreated with DMSO or SB203580, SB202190, PD169316, SB239063, or SKF86002 for 30 min, then LPS was added for 6 h and the G-CSF mRNA levels were measured by RT-qPCR. As shown in Fig. 2a, pretreatment with SB203580, SB202190, or PD169316 resulted in 3.6- to 4.7-fold increase, and SKF86002 pretreatment resulted in a 1.8-fold increase, in the G-CSF mRNA levels compared to LPS-treated cells; however, SB239063 pretreatment had no effect. Similar results were also obtained using human THP-1 cells (Fig. 2b). The inhibitory effect of these inhibitors and the lack of effect of the inactive SB203580 analogue SB202474 on LPS-induced p38 MAPK activation in THP-1 cells was confirmed by Western blot analysis of phosphorylation of HSP-27, a downstream effector of p38 MAPK (Fig. 2c). Thus, these results show that not all p38 MAPK inhibitors increase LPS-induced the G-CSF mRNA levels, suggesting that the SB203580-induced enhancement of the LPS-induced increase in the G-CSF mRNA levels might be unrelated to its effect of inhibiting p38 MAPK activity. We therefore examined whether SB203580 induced an increase in the G-CSF mRNA levels in cells p38 MAPK was not activated, i.e. in cells in which not treated with LPS. As shown in Fig. 3a, no p38 MAPK phosphorylation was detected in untreated RAW264.7 cells, but was detected after 15 min of LPS treatment and reached a maximum at 30 min. Although the G-CSF mRNA levels were low in untreated RAW264.7 cells, it could be detected after 35 cycles of RT-PCR and, as shown in Fig. 3b, SB203580 treatment for various times in the absence of LPS resulted in a 3-fold increase in the G-CSF mRNA levels starting after 4 h of treatment and lasting at least till 8 h. Since p38α MAPK is the major isoform, we then investigated whether it was involved in the SB203580-induced enhancement of the LPS-induced increase in the G-CSF mRNA levels using p38α MAPK knockdown in RAW264.7 cells by infection with a lentivirus carrying a specific shRNA targeting p38α MAPK or a lentivirus carrying luciferase shRNA as a negative control. Figure 4a shows that shRNA-mediated p38α MAPK knockdown resulted in an 80 % decrease in levels of p38α MAPK protein. The p38α MAPK and control knockdown cells were then pretreated with SB203580 for 30 min and treated with LPS for 6 h, then the G-CSF mRNA levels were measured by RT-qPCR. As shown in Fig. 4b, SB203580 induced a significant approximately 4-fold increase in the G-CSF mRNA levels in both LPS-treated p38α MAPK knockdown cells and control knockdown cells (Luc). The results support the above findings that the SB203580-induced enhancement of the LPS-induced increase in the G-CSF mRNA levels is independent of p38α MAPK.

### The SLDE in the 3’UTR is essential for SB203580-induced enhancement of the LPS-induced increase in the G-CSF mRNA levels

**Fig. 5:**
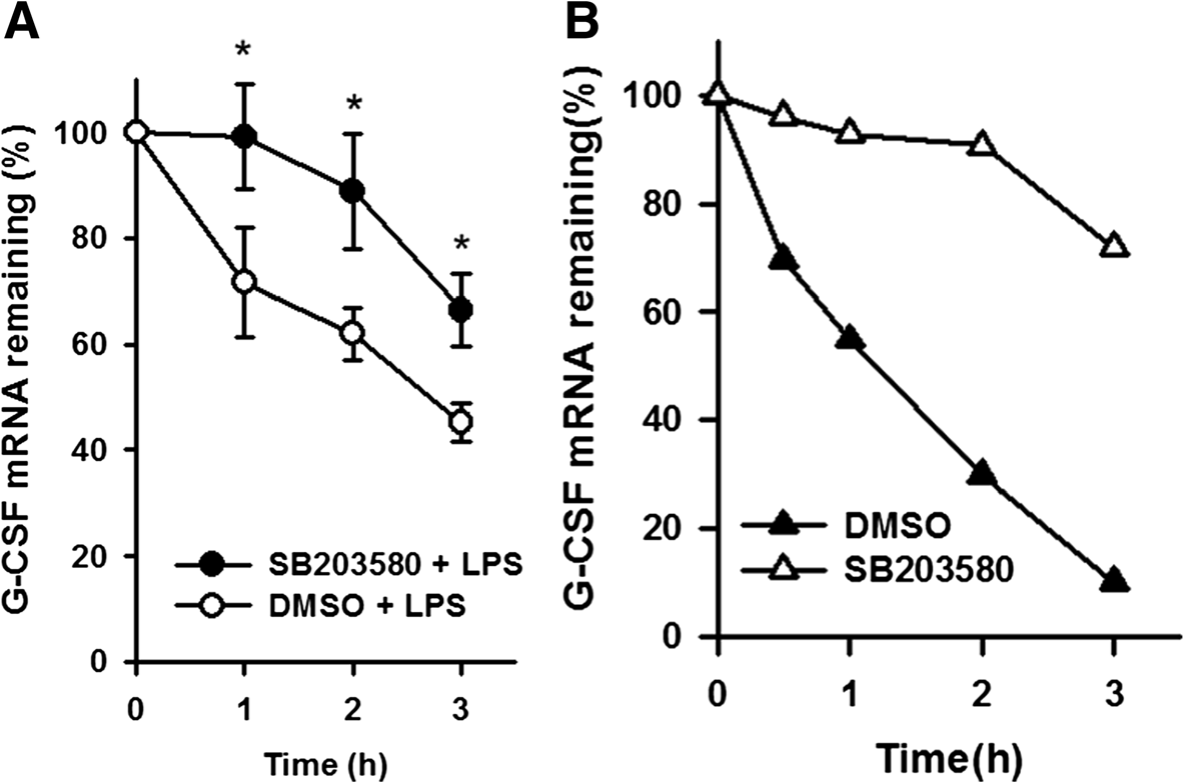
SB203580 slows G-CSF mRNA degradation in RAW264.7 macrophages. **a** RAW264.7 cells were pretreated for 30 min with DMSO or 10 μM SB203580, then 100 ng/ml of LPS was added and incubation continued for 5 h, then the cells were treated with actinomycin D (4 μg/ml) for 0, 1, 2, or 3 h. The G-CSF mRNA levels were determined by RT-qPCR, normalized to GAPDH mRNA levels, and expressed relative to the value at time 0, set as 100 %. The results are the mean ± SD for three independent experiments. *p < 0.05, compared to cells not treated with SB203580 at the corresponding time point. **b** RAW264.7 cells were pretreated for 30 min with DMSO or 10 μM SB203580, then actinomycin D (4 μg/ml) was added for 0, 0.5, 1, 2 or 3 h. Then the G-CSF mRNA levels were determined by RT-qPCR and normalized to GAPDH mRNA levels and expressed relative to the value at time 0 (relative value =100 %). The results are the mean for two independent experiments

**Fig. 6:**
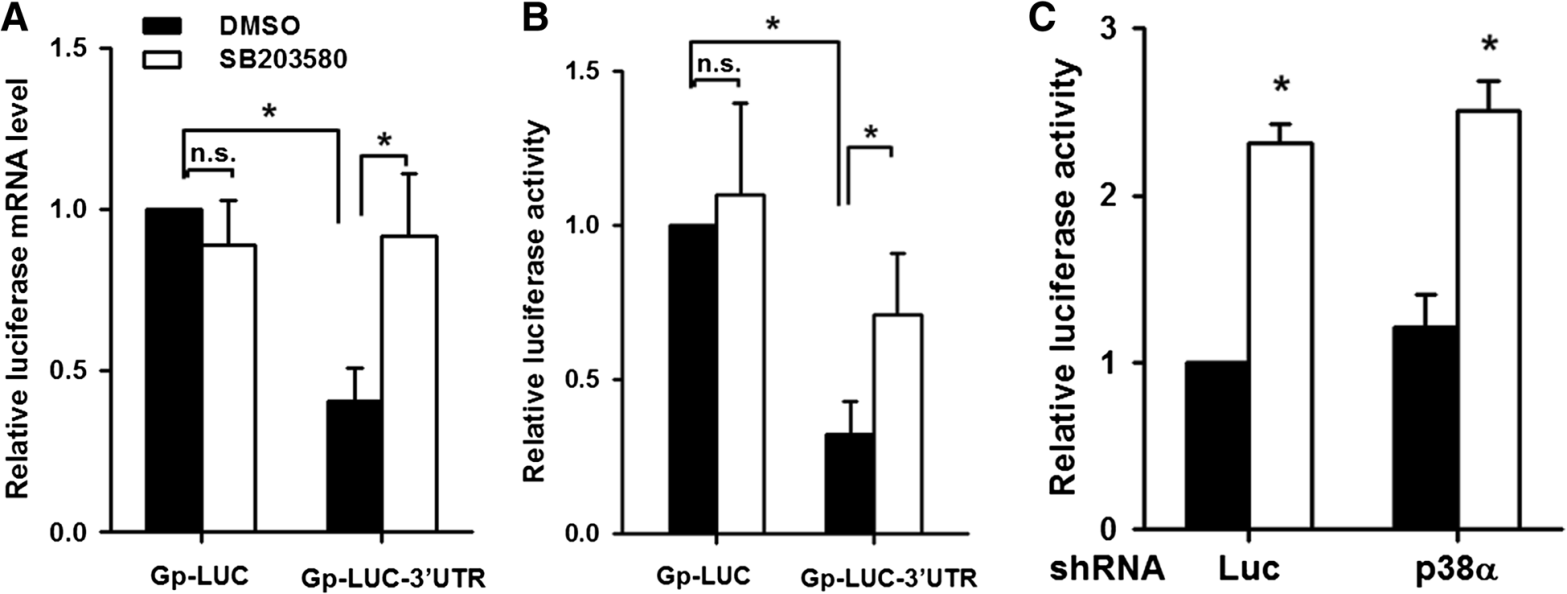
SB203580 induces the luciferase mRNA and activity in RAW264.7 cells transfected with Gp-LUC-3’UTR. (**a** and **b**) RAW264.7 cells were co-transfected with reporter plasmid (Gp-LUC or Gp-LUC-3’UTR) and control plasmid (phRLTK), then, 4 h later, were treated with DMSO or 10 μM SB203580 for 20 h. (**a**) Photinus luciferase mRNA levels were determined by RT-qPCR, normalized to Renilla luciferase mRNA levels, and expressed relative to the value in Gp-LUC-transfected cells treated with DMSO, set as 1. (**b**) Photinus and Renilla luciferase activities were measured and Photinus luciferase activity normalized to Renilla luciferase activity and expressed relative to the value in the Gp-LUC-transfected cells treated with DMSO, set as 1. (**c**) p38α MAPK knockdown cells and control knockdown cells (Luc shRNA) were co-transfected with Gp-LUC-3’UTR and phRLTK and treated as described above, then luciferase activities were determined as in (**b**) and expressed relative to the value in the DMSO-treated Luc controls, set as 1. The values are the mean ± SD for three independent experiments. *p < 0.05 as indicated. n.s., not significant

Since the 3’UTR of G-CSF mRNA contains several AUREs and an SLDE, both of which are mRNA stabilizing/destabilizing elements, it was possible that the SB203580-induced enhancement of the LPS-induced increase in the G-CSF mRNA levels was due to an increase in the stability of mRNA. To examine the effect of SB203580 on the stability of G-CSF mRNA, the G-CSF mRNA levels were determined by RT-qPCR in cells pretreated with DMSO or SB203580 for 30 min and subsequent LPS treatment for 5 h, followed by treatment with 4 μg/ml of actinomycin D for 0–3 h. As shown in Fig. 5a, in DMSO/LPS-treated cells, actinomycin D treatment resulted in a decrease in the G-CSF mRNA levels with a half-life of 2.7 h, while, in SB203580/LPS-treated cells, the half-life of G-CSF mRNA was approximately 3.7 h. In the absence of LPS, degradation of G-CSF mRNA was markedly slower in cells treated for 30 min with SB203580 than in those treated with DMSO when subsequently treated with actinomycin D (Fig. 5b). Together, these results show that SB203580 increases the stability of G-CSF mRNA. To determine whether the G-CSF 3’UTR was involved in SB203580-mediated mRNA stabilization, we first constructed a control reporter plasmid, Gp-LUC, which containing the luciferase gene driven by the GAPDH promoter, then cloned the 3’UTR from mouse G-CSF 3’ to the luciferase gene in the Gp-LUC plasmid to create a chimeric Gp-LUC-3’UTR reporter that could transcribe the luciferase mRNA containing the G-CSF 3’UTR. RAW264.7 cells were transfected with Gp-LUC or Gp-LUC-3’UTR, then, at 4 h after transfection, were incubated with DMSO or SB203580 for 20 h. In the absence of SB203580, the luciferase mRNA levels were significantly lower in cells transfected with Gp-LUC-3’UTR than in those transfected with Gp-LUC, and SB203580 treatment had no effect on the luciferase mRNA levels (Fig. 6a) or luciferase activity (Fig. 6b) in Gp-LUC-transfected cells, but resulted in a 1-fold increase in both the luciferase mRNA levels (Fig. 6a) and activity (Fig. 6b) in Gp-LUC-3’UTR-transfected cells. These results suggest that the 3’UTR of G-CSF mRNA may be involved in SB203580-induced mRNA stabilization, and show that the changes in luciferase activity paralleled those in the luciferase mRNA levels. Gp-LUC-3’UTR was also transfected into p38α MAPK knockdown cells or control knockdown cells, which were then treated as above with DMSO or SB203580 for 20 h and, as shown in Fig. 6c, SB203580 treatment resulted in a 1.3-fold increase in luciferase activity in both the p38α MAPK knockdown or control knockdown cells. This result supports our finding that SB203580 increases the G-CSF mRNA levels by an increase in its stability, probably mediated by the 3’UTR of G-CSF mRNA and independently of p38α MAPK activity.

**Fig. 7:**
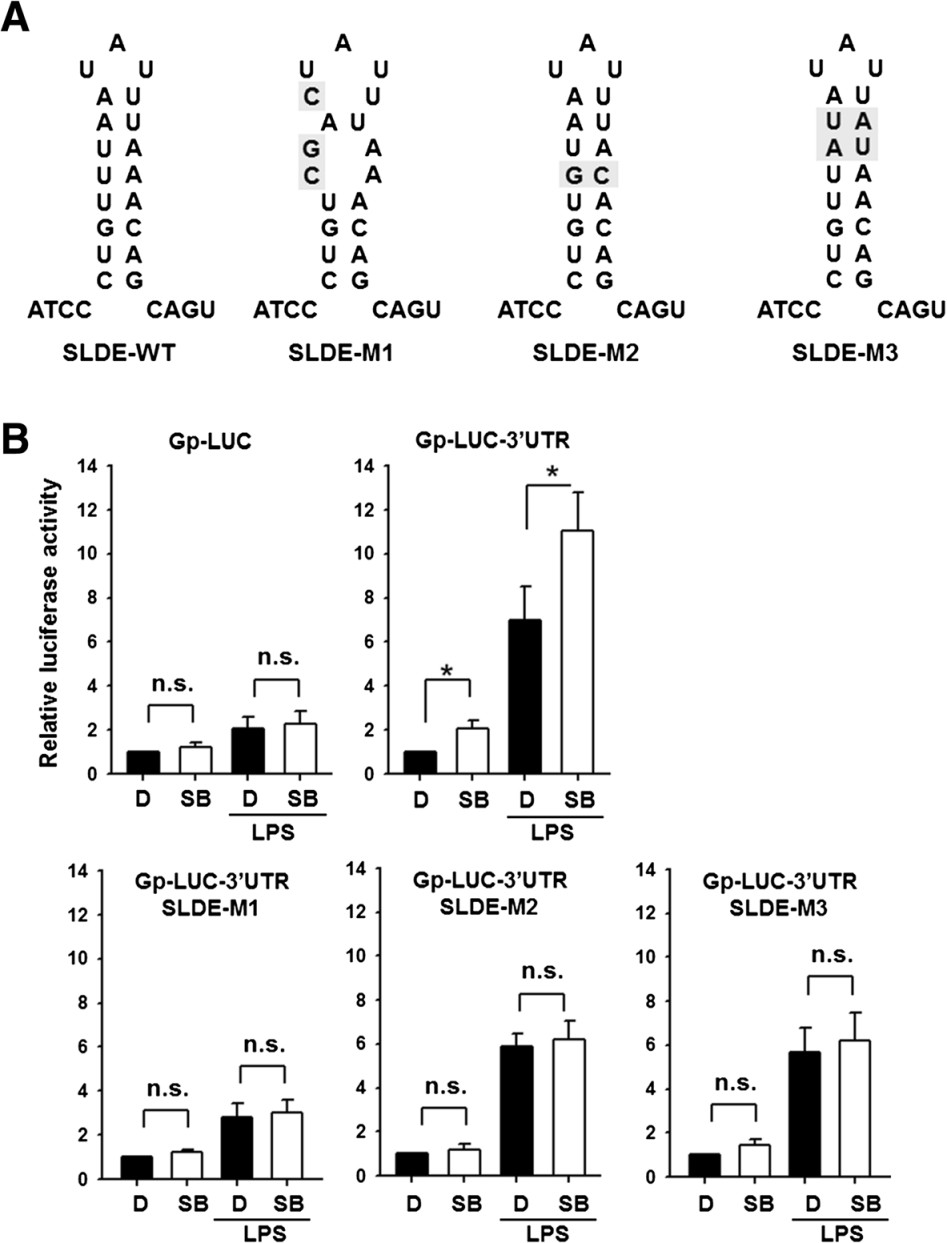
Mutation of the SLDE abolishes the SB203580-induced increase in luciferase activity in cells transfected with Gp-LUC-3’UTR. **a** The sequence and structure of the wild-type SLDE (WT) in the 3’UTR of G-CSF and the three SLDE mutants (M1, M2, and M3). **b** RAW264.7 cells were co-transfected with one of the reporter plasmids (Gp-LUC, Gp-LUC-3’UTR, or Gp-LUC-3’UTR mutants one to three) and control plasmid (phRLTK), then, 4 h later, were either treated with DMSO (D) or 10 μM SB203580 (SB) for 20 h (*left 2 columns*) or were pretreated for 30 min with DMSO or 10 μM SB203580, then LPS was added and the cells incubated for a further 20 h (*right 2 columns*), then the Photinus and Renilla luciferase activities were measured. The Photinus luciferase activity was normalized to the Renilla luciferase activity and expressed relative to the value for the DMSO-treated control for the same reporter, set as 1. The values are the mean ± SD for three independent experiments. *p < 0.05, compared to the corresponding DMSO-treated control. n.s., not significant

We then tested whether SB203580 pretreatment also induced an increase in luciferase activity in LPS-treated Gp-LUC- or Gp-LUC-3’UTR-transfected cells by treating the cells either with DMSO or SB203580 for 20 h or with these reagents for 30 min before and during incubation with LPS for 20 h. As shown in the top two panels of Fig. 7b, in the presence of LPS (two right columns in each panel), SB203580 increased luciferase activity by 110 % in cells transfected with Gp-LUC-3’UTR (right panel), but did not increase luciferase activity in cells transfected with Gp-LUC (left panel). To evaluate the role of the SLDE in the 3’UTR in the SB203580-induced increase in luciferase activity, we generated three Gp-LUC-3’UTR SLDE mutants, SLDE M1, M2, and M3 (Fig. 7a). Mutant M1 was constructed so as to disrupt the stem by mutating UUAA nucleotides on one side of the stem to CGAC, whereas the stem structure was retained in mutants M2 and M3, but a U-A base pair in the stem was replaced by a G-C base pair in M2 and two consecutive U-A and A-U base pairs were exchanged in M3. These reporter plasmids were transfected into RAW264.7 cells and the transfected cells were then incubated as above. The bottom 3 panels in Fig. 7b show that all 3 SLDE mutants lost the ability to response to SB203580 treatment in the absence or presence of LPS, while SLDE mutants two and three in which the stem structure was retained, but not mutant one in which it was destroyed, were still able to respond to LPS treatment. These results further support the idea that the SLDE in the 3’UTR of G-CSF mRNA is critical for the increase in the G-CSF mRNA levels induced by SB203580.

## Discussion

This study shows that SB203580 induced an increase in both basal and LPS-induced the G-CSF mRNA levels and that this effect was also seen in p38α MAPK knockdown RAW264.7 macrophages. Our results show that SB203580 stabilizes G-CSF mRNA and that this process requires the SLDE in the 3’UTR, but is independent of the inhibitory effect of SB203580 on p38α MAPK activity. Despite the fact that SB203580 is widely used as a p38 MAPK inhibitor, several biochemical studies have shown that it is also involved in many p38 MPAK-independent cellular activities [13–15]. This could be due to the facts that SB203580 inhibits several other protein kinases [15, 16] and induces activation of several others [17, 18]. Some SB203580-mediated cellular effects, such as inhibition of the 2,3,7,8-tetrachlorodibenzo-p-dioxin-induced increase in CYP1A1 mRNA levels and of α-melanocyte stimulating hormone-induced melanogenesis and spontaneous melanin synthesis, can be seen in cells treated with other pyridinyl imidazole inhibitors and also with SB202474, an SB203580 analog that does not inhibit p38 MAPK activity [13, 14]. These results suggested that all pyridinyl imidazoles might have these p38 MAPK inhibition-independent effects. However, in the present study, we found that SB203580, SB202190, and PD169316 were all effective in increasing the G-CSF mRNA levels, while SB239063 and SB202474 had no such effect, showing that only certain pyridinyl imidazole compounds can induce an increase in the G-CSF mRNA levels. Analysis of the chemical structures of these compounds suggested that the phenyl ring at the C-2 position and the 4-fluorophenyl group at the C-4 position of the imidazole ring are indispensable. However, the precise structure of compounds that can increase the G-CSF mRNA levels remains to be established.

The present study showed that SB203580 significantly increased the stability of G-CSF mRNA. Analyses using the luciferase-G-CSF 3’UTR chimeric reporter gene demonstrated that insertion of the 3’UTR downstream of luciferase resulted in downregulation of the luciferase mRNA and an increase in the luciferase mRNA levels in response to SB203580 treatment. Since luciferase transcription in both the Gp-LUC and Gp-LUC-3’UTR reporter constructs was driven by the GAPDH promoter, the decrease in the luciferase mRNA levels in cells transfected with Gp-LUC-3’UTR was probably due to destabilization of the luciferase mRNA caused by the 3’UTR from G-CSF mRNA. Although the AURE is the most common destabilizing element in mRNA, Brown et al. [6] demonstrated that the AUREs in G-CSF mRNA do not act as destabilizing elements in 5637 bladder carcinoma cells and identified the SLDE as a destabilizing element that functions independently of the AUREs. In this study, we mutated the sequence of the SLDE while leaving the AUREs intact and found that the ability of SB203580 to increase luciferase activity was abolished and, since the change of luciferase activity in paralleled the change in the luciferase mRNA levels, this suggests that the SLDE, but not AUREs, are responsible for the SB203580-induced increase in the stability of G-CSF mRNA. The precise mechanism is not known for that AUREs is not functional in G-CSF mRNA, the function of AUREs might be masked by the SLDE or by other unknown mechanism(s). The SLDE has been reported to increase the rate of mRNA deadenylation, and the sequence within the stem was found not to be critical for the destabilizing effect as long as the stem structure was maintained [4]. However, our results suggest that both the structure and the sequence of the stem are critical for G-CSF mRNA induction by SB203580. Taken together, our results suggest that the SLDE functions as a destabilizing element in untreated macrophages and becomes a stabilizing element in SB203580- and/or LPS-treated macrophages.

Cancer patients usually develop neutropenia after chemotherapy or radiation therapy [19] and such patients are at risk of bacterial infections, so an effective and low cost therapy that can accelerate the recovery of neutrophil numbers is important. This effect can be achieved in neutropenia patients by injection of recombinant G-CSF [20]; however, this requires subcutaneous or intravenous injection and is expensive. Because of this, efforts have been made to identify small molecular weight compounds that can be delivered at low cost as an effective therapy. A small synthetic compound, SCH 14988 (a pyridinyl-naphthyridinyl-sulfonyl acetamide), has been shown to specifically stimulate G-CSF production in vitro and in vivo [21]. In addition, Aoki et al. [22] identified a stimulatory monoclonal antibody (mAb 3-4H7) from autoimmune mice that selectively stimulates G-CSF gene expression in mouse macrophages. The mechanism(s) by which SCH 14988 and mAb 3-4H7 stimulate G-CSF production is unclear, but post-transcriptional regulation has been suggested in the case of SCH 14988-stimulated G-CSF production [21]. Our study demonstrated that a small molecular weight compound, SB203580, can increase the G-CSF mRNA levels through a post-transcriptional mechanism by increasing the stability of mRNA. Our results show that the development of small molecular compounds that increase the stability of G-CSF mRNA by acting on a stabilizing element in the 3’UTR could be a novel approach to stimulate G-CSF production.

p38 MAPK is a major phosphoprotein activated by LPS and several inflammatory cytokines [7, 23]. After phosphorylation/activation, p38 MAPK activates downstream mediators that upregulate the production of multiple pro-inflammatory cytokines, including TNFα, IL-1β, IL-6, and IL-8, and the induction of key inflammatory enzymes, including COX-2 and inducible nitric oxide synthase [7, 23]. p38 MAPK has therefore been suggested as a therapeutic target for treatment of inflammatory diseases [23]. However, when various p38 MAPK inhibitors were used in two similar acute lung inflammation rodent models, conflicting results were obtained. Arcaroli et al. [24] demonstrated that systemic inhibition of p38 MAPK by SB203580 resulted in increased pulmonary neutrophil accumulation in LPS-induced acute lung inflammation, whereas other workers found that SB239063, another p38 MAPK inhibitor, significantly reduced pulmonary neutrophil infiltration [25, 26]. Since G-CSF is a key regulator of the production of neutrophils, these conflicting results might be due to SB203580 inducing G-CSF production, leading to neutrophil accumulation, whereas SB239063 might have no effect on G-CSF production. G-CSF levels were not measured in these studies, so it would be interesting to investigate the effects of SB203580 and SB239063 on G-CSF production and pulmonary neutrophil infiltration in LPS-induced acute lung injury.

## Conclusions

We have shown that SB203580, a p38 MAPK inhibitor, increases the G-CSF mRNA levels by increasing the stability of mRNA and that this effect is independent of its inhibitory effect on p38 MAPK activity. Our results also suggest that the SLDE in the 3’UTR of G-CSF mRNA functions as a destabilizing element in the absence of treatment and becomes a stabilizing element in response to SB203580 treatment. The results of this study provide a potential approach for boosting endogenous production of G-CSF during neutropenia. Different effects of different pyridynyl imidazole compounds on G-CSF production might explain the conflicting results obtained using these compounds in different anti-inflammation studies. Exactly how the SLDE is involved in increasing the stability of G-CSF mRNA is not known at this time, and further studies are needed to clarify the mechanism.

## Abbreviations

AURE: Adenosine uridine-rich elements
BMDMs: Bone marrow-derived macrophages
COX-2: Cyclooxygenase-2
DMEM: Dulbecco’s modified eagle’s medium
DMSO: Dimethyl sulfoxide
FBS: Fetal bovine serum
G-CSF: Granulocyte colony-stimulating factor
IL-1β: Interleukin-1 beta
LPS: Lipopolysaccharide
MAPK: Mitogen-activated protein kinases
qPCR: Quantitative PCR
SLDE: Stem–loop destabilizing element
shRNA: Short hairpin RNA
3’UTR: 3’ untranslated region
